# “Why should care workers be any different from prison workers?” A qualitative study of second-hand smoke exposure during home-care visits and potential measures to eliminate exposure

**DOI:** 10.1093/annweh/wxae069

**Published:** 2024-08-19

**Authors:** Rachel O’Donnell, Ruaraidh Dobson, Sean Semple

**Affiliations:** Institute for Social Marketing and Health, Faculty of Health Sciences and Sport, University of Stirling, Stirling, FK9 4LA, United Kingdom; Institute for Social Marketing and Health, Faculty of Health Sciences and Sport, University of Stirling, Stirling, FK9 4LA, United Kingdom; Trilateral Research Ltd, One Knightsbridge Green, London SW1X 7QA, United Kingdom; Institute for Social Marketing and Health, Faculty of Health Sciences and Sport, University of Stirling, Stirling, FK9 4LA, United Kingdom

**Keywords:** home-care workers, qualitative, second-hand tobacco smoke

## Abstract

**Introduction:**

Despite world-leading measures in place to protect employees from second-hand smoke exposure in workplaces in the United Kingdom, workers who deliver health and social care in private homes remain unprotected legally in this setting from second-hand smoke exposure (SHS).

**Methods:**

Fourteen individuals took part in either an in-depth telephone interview (*n* = 11) or an online focus group discussion (*n* = 3), including home-care workers (*n* = 5) and managers (*n* = 5) based in Lanarkshire (Scotland) and local/national policy makers (*n* = 4). Participants were asked about the extent to which exposure to SHS is an issue during home visits and possible additional measures that could be put in place to eliminate exposure.

**Results:**

Participants highlighted the difficulties in balancing the provision of care in a person’s own home with the right of workers to be able to breathe clean air and be protected from SHS. Current strategies to reduce staff exposure to SHS during home visits were often reported as inadequate with SHS not a hazard considered by managers beyond protecting pregnant staff or those with pre-existing respiratory conditions such as asthma. Simple respiratory protective equipment (as used during the COVID-19 pandemic) was rightly identified as being ineffective. Methods such as nicotine replacement therapy and e-cigarettes were identified as potential ways to help people who smoke achieve temporary asbstinence prior to a home visit.

**Conclusion:**

Implementing appropriate and proportionate measures to protect home-care workers from the harms posed by SHS should be a priority to help protect the health of this often overlooked occupational group.

**What’s Important About This Paper?**

The findings of this study highlight an inconsistency in Article 8 of the World Health Orgnisation Framework Convention on Tobacco Control, which provides protection from second-hand smoke in indoor workplaces in many countries, but does not cover people working in private homes. This requires addressing with clear guidelines to provide protection for people working in domestic settings from the harms of second-hand smoke, including domiciliary home-care workers—a largely female group of workers.

## Introduction

Second-hand tobacco smoke (SHS) causes approximately 1.2 million deaths globally per year through cancer, respiratory, and cardiovascular condtions, including heart attacks and stroke (World Health Organisation 2020). Exposure to SHS in the workplace has been a major occupational health issue since it was first identified as being associated with an increased risk of lung cancer in 1981 (Trichopoulos et al. 1981). The United Kingdom has been a global leader in introducing comprehensive measures to protect the population from exposure to SHS: the proportion of non-smoking adults who show biological evidence of exposure to SHS has fallen from 83% to 19%. It is likely that most of this reduction is the result of legal restrictions in workplaces and social settings (Semple et al. 2019a). Historically smoke-free measures were introduced in the United Kingdom from the perspective of population health protection with occupational health benefits often seen as incidental. In the United Kingdom, developments over the past 15 years have seen almost all workplace settings, ranging from the hospitality sector (2006/7) through to prisons (2018) (Semple et al. 2019b), introducing legislation and policy to explicitly protect workers from SHS. As a result, levels of salivary cotinine (a marker of nicotine exposure) in non-smoking adults have decreased by 97% in the past 20 years (Semple et al. 2019a).

This success, perhaps one of the most major achievements in protecting the health of workers in the United Kingdom since the turn of the century, has not however applied to all workers. Those involved in working to deliver health and social care in private homes find themselves in settings where there are no legal protections in terms of SHS or restrictions on smoking. A recently developed job-exposure matrix suggests that approximately 1.9% of the UK workforce experienced daily (and for more than 1 h per day) exposure to SHS indoors in a poorly ventilated environment (Dobson et al. 2021). The majority of these workers all work regularly in other people’s homes with domiciliary health and care workers (DHCWs) making up the majority of this workforce. DHCWs are a large and growing group of over 600,000 workers (UK Home Care Association 2016) in the United Kingdom who perform day-to-day work tasks that involve spending time in patients’ homes where smoke-free regulations do not apply. Recent survey data from Scotland indicated that 74% of DHCWs reported exposure to SHS, with 50% of those reporting “daily” exposure (Dobson et al. 2023).

Explicit policies and guidance to manage domiciliary workers’ exposure to SHS are rare, overlooked, and often out-dated (Royal College of Nursing 2006). For example, the Royal College of Nursing (RCN) advice issued in 2006 advises those receiving care at home not to smoke for one hour prior to a visit: guidance that contrasts with public health messaging about how long SHS remains in household air (Semple and Latif 2014).

A recent international review identified little consideration of DHCW’s exposure to SHS during their work activity, and a need for development of policies and measures to protect those whose jobs involve providing assistance in domestic settings (Angus and Semple 2019). DHCWs require protection from SHS in the same way that occupational health first intervened to protect the health of airline flight attendants in the 1980s (Repace 2004). There is a clear need to find solutions and policy measures to protect care workers from the harms of SHS exposure.

This research used qualitative methods to explore the views and experiences of those who manage and have responsibility for policies relating to DHCWs, with a particular focus on potential measures that could be implemented to protect workers from SHS exposure.

## Methods

### Design and sample

Data collection (from May 2021 to February 2022) included 10 in-depth telephone interviews with DHCWs (*n* = 5) and managers (*n* = 5) based in NHS Lanarkshire (Scotland). In addition, one in-depth telephone interview and one online focus group discussion (with 3 participants) were conducted with local/national policy stakeholders (*n* = 4).

All DHCWs/managers who were approached to take part in interviews had previously participated in large-scale survey work and/or personal exposure monitoring as part of this study (Dobson et al. 2023). All policy stakeholders were identified via existing contacts of the research team. Each individual received an information sheet with study details and provided consent to participate and be audio-recorded. We aimed to ensure participants included a mix of those who work for the National Health Service (NHS) in Scotland and local authority bodies.

### Procedure

Interviews/discussion groups were conducted using a semi-structured interview schedule/topic guide (see supplementary material) developed during team discussions. Issues explored included (i) the extent to which exposure to SHS is an issue during home visits, (ii) views regarding current guidelines, and (iii) possible additional measures that could be put in place to eliminate SHS exposure during home visits. Ethical approval for the study was received from the NHS Cambridge East ethics committee (20/EE/0121).

### Analysis

Discussions lasted between 46 min and 1 h 7 min (median = 57 min). Using NVivo 12, two team members coded anonymised transcripts against a set of categories (see supplementary material) created using deductive (reviewing research questions) and inductive approaches (reading transcripts). Each category was developed iteratively, with ongoing refinements made while re-examining the data and based on review by both coders. Interim analysis findings were refined and finalised in discussion with study team members. Additional illustrative quotes for key themes presented in the Results are included in supplementary material.

## Results

### Balancing the needs and rights of service users and care workers

Managers and care workers often spoke of a perceived conflict between the rights of patients/carers to smoke in their own home, and the rights of DHCWs to clean air within their workspace:

“I think sometimes people can become very blinkered with saying, ‘well, we’ve got a service to provide’, instead of actually thinking, ‘we’ve got a duty of care towards our staff.’” (Participant 3, Manager)

Several participants noted that some individuals who receive care at home are amongst the most vulnerable in the local community. In addition, in scenarios such as the provision of end-of-life care, one manager felt that DCHWs have few options: “It’s not like you can refuse to go in, or ask the care to be delivered somewhere else.” (Participant 2, Manager)

The rights of service users often centred around the fact that services are delivered in individual’s homes. Several participants noted this could lead to challenges in speaking with patients about refraining from smoking prior to/during home visits. To overcome this challenge, one manager noted potential methods of negotiation:

“I say ‘look, I’m a really bad asthmatic…do you mind if I open your front and back door just to let some of air through?’ No-one has ever refused me…I have passed that [tip] on to staff I’ve worked with over the years.” (Participant 3, Manager)

### Current strategies to reduce staff exposure to SHS during home visits

A few managers noted that risk assessments were conducted with a view to protecting specific sub-groups of workers from SHS exposure, including pregnant women and indivduals with existing respiratory conditions:

“If staff let us know that they’re pregnant, then we would complete a risk assessment…and we would be highlighting that they shouldn’t be around second-hand smoke…the same as if we had someone on the team who has really bad asthma or any respiratory condition.” (Participant 2, Manager)

However, this practice did not appear to be standardised, and one manager noted that exposure to SHS was not classed as a hazard in their risk assessment guidance:

“It doesn’t have anything specifically to do with exposure to second-hand smoke, it’s all about mental fatigue, exposure to infection, biological agents…” (Participant 4, Manager)

Participants agreed that the 2006 RCN guidance advising patients and their carers not to smoke in the home for up to 1 h before a visit does not adequately protect staff. However, most felt it is unfeasible to expect individuals to refrain from smoking for up to 5 h, in line with current public health messaging, without additional support:

“Five hours is a lifetime to some people. They would probably throw their hands up in horror if you said, ‘don’t smoke for five hours’.” (Participant 8, Care worker)

Given this study was conducted during the COVID-19 pandemic, when DHCWs (like many other workers) were required to wear respiratory protective equipment (RPE) to reduce their risk of COVID-19 infection, the extent to which RPE would offer adequate protection was also discussed. It is worth noting that most RPE used by DHCWs during the pandemic would provide no protection from the fine Particulate Matter (PM_2.5_) generated by smoking. Most participants shared this view:

“The small blue things [masks] that you see us all wearing, they wouldn’t have any benefit whatsoever. Not even for a minute.” (Participant 3, Manager)

### Supporting temporary abstinence from smoking in the home prior to/during home visits

Participants discussed views on the use of nicotine replacement therapy (NRT) products and e-cigarettes for temporary abstinence from smoking in the home, as a means of eliminating DHCWs’ exposure to SHS. They held generally positive views on the temporary use of NRT products, with one manager suggesting some Health Board areas already use this approach in the context of home visits to provide palliative care. They went on to suggest:

“It [NRT use] could make a big difference...They [patients] may actually realise, ‘well, I don’t need to have a cigarette.’ And it could potentially ease a lot of the concerns that nurses have about going into smoky environments.” (Participant 4, Manager)

From a policy perspective, the logistics of supplying NRT, ensuring effective use of products, and the potential costs were also discussed, but the general consensus was that using NRT for this purpose was a potential solution:

“Few people would argue against using nicotine replacement therapy for that [purpose]” (Participant 14, Policy stakeholder).

Participants were generally more cautious about the use of e-cigarettes as a means of temporary abstinence from smoking in the home. Several voiced concerns regarding a lack of knowledge about the health impacts of e-cigarette use (“I don’t think any of us know enough about e-cigarettes” (Participant 1, Manager)). Others raised questions regarding the potential harms associated with exposure to second-hand aerosols from e-cigarettes:

“I wouldnae really want anybody to use an e-cigarette when I’m in [their home] because I don’t think there’s enough knowledge about what the effects are.” (Participant 9, Care worker)

A few policy stakeholders suggested this approach could be challenging to adopt at the NHS Board level, given current policy prohibits e-cigarette use in NHS hospital grounds, whilst others noted the broader potential benefits of e-cigarette use as a harm reduction tool for people who smoke.

## Discussion

All interviews with care workers and managers were held during the COVID-19 pandemic, at a time where home care workers were not routinely carrying out home visits and/or had been deployed into hospitals to help with community vaccination clinics. Their reflections were therefore based on experiences prior to March 2020.

The findings of this work add to the current evidence base regarding DHCW’s experiences of SHS exposure and highlight potential policy and practice solutions to address this significant issue.

Implementing appropriate and proportionate measures to protect DHCWs from the harms posed by SHS should be a priority to help protect the health of this often overlooked occupational group. Given the potential growth in this sector due to an ageing population in many high-income countries, there is a need to develop a policy framework that better protects DHCWs from the harms of SHS. Over 80% of home care workers are female (Skills for Care 2023) making this a gendered inequality issue in terms of occupational exposure to SHS.

There is a need for greater attention to this issue and development of a manifesto to tackle exposure to second-hand tobacco smoke among this group of workers. The focus of this current work has been on SHS but we also acknowledge that Thirdhand Smoke (THS) is likely to be a route of exposure to toxins from cigarette smoke as DHCWs touch surfaces and handle objects in smokers’ homes. The potential for dermal and ingestion uptake of nicotine and other tobacco toxins should be considered for these workers (Gorman Ng et al. 2012).

A shift in priorities among those involved in managing workers within this sector is required: accepting that SHS exposure is as important a hazard as manual handling/lifting, infectious disease, or chemicals. Debate, education, and consideration of the rights of DHCWs to breathe clean air at work are required: this will include media campaigns that highlight the need for society to protect those who care for us within our homes. Finally, Article 8 of the World Health Organisation Framework Convention on Tobacco Control currently provides protection from exposure to SHS in indoor workplaces in many countries; however, it does not cover people working in private homes. This is an inconsistency that needs to be addressed with clear guidelines to protect people working in domestic settings from the harms caused by SHS.

## Supplementary material

Supplementary material is available at *Annals of Work Exposures and Health* online.

wxae069_suppl_Supplementary_Material

## Data Availability

The anonymised data underlying this article may be shared on reasonable request to the corresponding author following review.
