# Supplementary material for: “Why should care workers be any different from prison workers?” A qualitative study of second-hand smoke exposure during home-care visits and potential measures to eliminate exposure
Source: Ann Work Expo Health. 2024 Aug 19;68(9):999–1003. doi: 10.1093/annweh/wxae069 (PMC11586273; doi:10.1093/annweh/wxae069)
Supplement: wxae069_suppl_Supplementary_Material [file wxae069_suppl_supplementary_material.pdf]

“Why should care workers be any different from prison workers?” A qualitative study of second-hand smoke exposure during home-care visits and potential measures to eliminate exposure.

Rachel O’Donnell (PhD) (1)

Ruaraidh Dobson (PhD) (1, 2)

Sean Semple (PhD) (1)

1. Institute for Social Marketing & Health, Faculty of Health Sciences and Sport, University of Stirling, Stirling, FK9 4LA, Scotland, UK

2. Trilateral Research Ltd, One Knightsbridge Green, London SW1X 7QA, England, UK

Supplementary Material: Interview schedules/topic guide, codebook and additional illustrative quotes for key themes presented

Protecting those who care for us: Interview schedule – Care workers

Interview/Participant number: ____ Date: ____

1. **Work role**

I'd like to ask you a couple of questions about your work, just to give us a bit of context:

a. What is your job title?
b. How long have you been working for the NHS/private company you currently work for?
c. What do you most enjoy about your job?
d. What are the key challenges you face in your role?

2. **Exposure to second-hand smoke during home visits**

a. How often do you enter clients' homes as part of your work? *E.g. Every shift, most days, less often than that?*
b. How often are you exposed to second-hand smoke in clients' homes? *E.g. Every day, most days, some days, not that often etc*
c. Who does client health/risk assessments? Is smoking considered as part of that? When you first go to a client's house, what information are you given about the client in advance? Would you be aware if they are a smoker before you enter their home for the first time?
d. To what extent do you feel this is a problem (a) for you (PROMPT: the smell v the health impact) and (b) for the wider domiciliary care workforce? How do your fellow colleagues feel about this issue? To what extent is it discussed? Has it ever been raised as an issue in team meetings for example? If so, what were your managers responses?
e. Do you ever smell cigarette smoke when you enter a client's home? How does this make you feel?
f. Do you think that clients/other household members ever smoke in the home in the hour or so *before* you home visit?
g. Does this concern you?

   If yes:
   - Do you feel comfortable to address this directly with clients?
   - Have you ever spoken with clients/other household members about them refraining from smoking in the home before your visit? If yes, how did they react to this?

h. Have you experienced clients/other household members smoking in the home *during* your home visit?

   If yes:
   - Do you feel comfortable to address this directly with clients?
   - Did/do you ever speak with clients/other household members about them refraining from smoking in the home during your visit? If yes, what was their reaction to this?

i. Have you ever specifically asked not to visit a client because of exposure to second-hand smoke? If so, are you able to tell me more about this? What was the outcome of this request?

j. Do clients/other household members routinely refrain from smoking for at least 1 hour before you arrive in their home?

   If yes:
   - Do you think this protects you from second-hand smoke exposure?

   If no:
   - What barriers do you think clients/other household members face in refraining from smoking in this way? Are there any additional supports that could be put in place to help them with this?

k. Has the Covid-19 pandemic changed clients' smoking behaviours before/during visits? For example, have you noticed clients smoking more/less in the home? Any other changes?

If not already discussed in relation to the questions above:

l. Do you have a pre-existing health condition that increases your vulnerability to second-hand smoke exposure during home visits? Do you feel adequately protected from second-hand smoke exposure during home visits?

m. Have you worked as a domiciliary care worker during pregnancy at any point? If yes, did you/do you feel adequately protected from second-hand smoke exposure during home visits?

3. **Current guidelines on exposure to second-hand smoke during home visits**

The Royal College of Nursing advice issued in 2006 advises those receiving care at home not to smoke for one hour prior to a visit. You may be aware of recent evidence, used in the Scottish Government's 'Take it Right Outside' campaign, which suggests that second-hand smoke can linger in the air for up to 5 hours.

a. Do you know if your employer developed any additional guidance or policy to protect you? If so, what additional guidance/policy is in place? Is there any training/support in place for raising difficult conversations?

b. Is there anything more your employer could do to ensure that you are better protected from second-hand smoke exposure at work?

c. How do you feel existing policies compare with those in place for staff in other work roles (i.e. hospitality, prisons etc)?

4. There are no plans to introduce either of the following measures – we are just interested in your views on them:

   a. What are your views on clients using nicotine replacement therapy (NRT) during your visit, instead of smoking? How would you feel about giving them NRT to use on arrival?

   b. How would you feel about clients using e-cigarettes during your visit, instead of smoking?

   c. Do you feel that wearing a mask during home visits would adequately protect you from second-hand smoke?

   d. Ideally, what measures would you like to see put in place moving forward? Can you identify and specific pros and cons associated with this approach?

Are there any other issues related to second-hand smoke exposure in the home that you would like to raise? Or any other questions before we close?

[END]

Protecting those who care for us: Interview schedule – Managers

Interview/Participant number: ____ Date: ____

1. Work role

a. What is your job title?
b. How long have you been working for the NHS/private company you currently work for?
c. How many in-home care workers do you work with in your role? How many does your organisation employ?
d. Could you outline briefly the key responsibilities of your role?
e. What do you most enjoy about your job?
f. What are the key challenges you face in your role?

2. Existing policies on second-hand smoke

a. Do many of the clients/patients that your staff work with smoke?
b. Have you had care workers express concern about entering homes where people smoke?
c. What policies/guidance does your organisation have in place on care workers' second-hand smoke exposure? How often is it reviewed? When was it last reviewed?
d. How was this policy/guidance developed? Was it developed in conjunction with staff? By senior staff/Board members?
e. How do you communicate those policies to workers?
f. How do you communicate relevant policies to clients/patients? Do you have leaflets/policy documents to give to them?
g. Are there any informal arrangements in place (in addition to official policies) to tackle this issue? *For example, assigning only smokers to work with clients/patients who smoke; ensuring staff who are pregnant/have pre-existing health conditions are not assigned to work with clients/patients who smoke.*

3. Future policy/guidance developments

The Royal College of Nursing advice issued in 2006 advises those receiving care at home not to smoke for one hour prior to a visit. You may be aware of recent evidence, used in the Scottish Government's 'Take it Right Outside' campaign, which suggests that second-hand smoke can linger in the air for up to 5 hours.

a. Do you feel you have enough information about the potential health harms associated with second-hand smoke exposure?
b. To what extent do you feel your current policy/guidance is working to adequately protect care workers from second-hand smoke exposure?
c. Do existing policies correctly balance the health needs of care workers with the care needs of clients/patients?
d. How do you feel existing policies compare with those in place for staff in other work roles (i.e. hospitality, prisons etc)?
e. Are there any ways you think existing policies could be improved?

4. There are no plans to introduce either of the following measures – we are just interested in your views on them:

a. What are your views on clients using nicotine replacement therapy (NRT) during home visits, instead of smoking?
b. What are your views on clients using e-cigarettes during home visits, instead of smoking?
c. What are your views on in-home care workers wearing a mask during home visits?

Are there any other issues related to second-hand smoke exposure in the home that you would like to raise? Or any other questions before we close?

[END]

Protecting those who care for us: Interview schedule/topic guide - Policymakers

Interview/Participant number: ____ Date: ____

1. Work role

I'd like to ask you a couple of questions about your work, just to give us a bit of context:

a. What is your job title?
b. How long have you been working for the NHS/private company you currently work for?
c. What do you most enjoy about your job?
d. Could you outline briefly the key responsibilities of your role?

2. Existing policies on second-hand smoke

a. What policies/guidance if any have you been involved in developing or delivering relating to care workers' second-hand smoke exposure? How often is it reviewed? When was it last reviewed?

b. What policies/guidance if any have you been involved in developing or delivering relating to care workers' second-hand smoke exposure? How often is it reviewed? When was it last reviewed?

c. How was this policy/guidance developed? Was it developed in conjunction with staff? By senior staff/Board members?

e. How do you or your organisation communicate those policies to workers or others?
f. If applicable, how do you communicate relevant policies to clients/patients? Do you have leaflets/policy documents to give to them?
g. What are the key process and factors to consider when implementing policies of this type? Possible prompts: how to engage clients constructively, role played by line mangers, workers support and training needs, ongoing system for monitoring and feeding back workers experiences etc
h. How do you implement and enforce these policies in practice?

3. Future policy/guidance developments

The Royal College of Nursing advice issued in 2006 advises those receiving care at home not to smoke for one hour prior to a visit. You may be aware of recent evidence, used in the Scottish Government's 'Take it Right Outside' campaign, which suggests that second-hand smoke can linger in the air for up to 5 hours.

a. To what extent do you feel that current policy/guidance is working to adequately protect care workers from second-hand smoke exposure?

b. Do existing policies correctly balance the health needs of care workers with the care needs of clients/patients? What about for specific groups of workers, i.e. those who are pregnant, those with existing health conditions?

c. How do you feel existing policies compare with those in place for staff in other work roles (i.e. hospitality, prisons etc)?

d. Are there any ways you think existing policies could be improved / lessons could be learnt from other work place policies?

4. There are no plans to introduce either of the following measures – we are just interested in your views on them:

a. What are your views on clients using nicotine replacement therapy (NRT) during home visits, instead of smoking?

   b. What are your views on clients using e-cigarettes during home visits, instead of smoking?
   c. What are your views on in-home care workers wearing a mask to protect them from second-hand smoke during home visits? Have these views changed since the start of the Covid pandemic?

5. **What do you think about the following statement: "Patients/clients should have the right to smoke in their own home."**

6. **And similarly, but from the other perspective, what does the following statement make you think: "How can you ask me, as a carer, to go into a home and breathe a known carcinogen every day at work?"**

7. **Thinking really broadly, and if you were starting from scratch, how would you design a policy to best protect the home care workforce from exposure to second-hand smoke? What would be your ideal approach?**

8. **Are there any other issues related to second-hand smoke exposure in the home that you would like to raise? Or any other questions before we close?**

[END]

Analysis Codebook

**1. Background**	**Participant characteristics disclosed at interview**
1a. Work roles and patterns, inc in-home contact	Current employer/sector, job title/role, career history/experiences, whether ever worked in clients homes/ in what capacity / how often in clients homes; how their work is organised - drive or walk between clients; attitudes towards their work, things about their work they enjoy/find challenging
1b. Health & smoking	Health conditions, vulnerabilities to SHS exposure; own smoking status/ history
**2. Exposure to second-hand smoke (SHS)**	
2a. In clients homes	Exposure to (or absence of) SHS in clients homes: current, past, frequency, trends/changes over time, accuracy
2b. In other settings	Employers offices/work base, travel between clients, work vehicles etc
2c. Impact of Covid-19 pandemic	On clients smoking behaviour and SHS exposure; work practices and patterns – social mixing; personal experiences of Covid and returning to work, impact of SHS on respiratory symptoms etc
**3. Workers smoking behaviour**	In clients homes, policy on smoking in clients homes; level of adherence, other factors effecting smoking at work, eg. too busy/not practical)
**4. Clients and other family members smoking behaviours**	Extent to which they smoke openly in front of workers; social context / interactions between family members; any spontaneous actions typically taken to moderate their behaviour/minimise workers (and other family members) exposure; perceptions of clients beliefs/position on smoking and SHS exposure; variations by type of client etc
**5. Attitudes and salience of SHS exposure**	In client's home and other settings; self and other work colleagues; how feel about clients smoking/being exposed at work; extent to which it is seen as a problem, how problem is characterised: health, smell etc; any variations by worker-type, eg. those with respiratory conditions, workers who smoking/vape etc
**6. Interactions with clients re their smoking, inc barriers & facilitators**	Prior awareness / pre-notification of clients and other householders smoking status, whether ever seek to reduce personal exposure, types of actions taken, effectiveness, challenges – ability to accurately monitor smoking behaviour/reliance on senses, how they feel about/willingness to raise the issue; strategies used; when do they broach the issue and why; whether ever refused to enter a clients home – why, reactions; useful learning re actions taken etc
**7. Related policy, guidance and management support**	Awareness of any policy re managing worker exposure (including 1 hour rule) / related health and safety / risk assessment issues; is this communicated to clients / consistently implemented; extent to which it is acknowledged / discussed with line managers; managers attitudes towards workers exposure; kinds of support put in place / actions taken; comparisons with support given in other settings; adequacy of current support; need for policy development - key areas; key challenges and barriers etc.
**8. Views (pros and cons) on specific policy areas & interventions**	
8a. Use of nicotine replacement therapies	Views on use of/giving out nicotine replacement therapy (NRT) during visits, instead of smoking
8b. Use of E-cigarettes & vaping	Feelings about client use of e-cigarettes during visits, instead of smoking

8c. Use of mask wearing	Feelings about mask wearing during home visits / adequacy of protection / new Covid norms etc
8d. Response to new evidence re smoke lingering for 5 hours	Awareness and response to recent evidence that second-hand smoke can linger in the air for up to 5 hours
8e. Pregnancy & SHS exposure	Feelings about / experiences of SHS exposure at work during pregnancy; management support / actions taken
**9. Miscellaneous**	Uncoded data of possible value

Additional ilustrative quotes for key themes presented

Balancing the needs and rights of service users and care workers

"I think sometimes people can become very blinkered with saying, 'well, we've got a service to provide', instead of actually thinking, 'we've got a duty of care towards our staff.' (Participant 3, Manager)

"*We're supporting increasing numbers of people with cognitive impairments, addiction issues...individuals who are frail or they're immobile. Um, so can be some real difficulties…"* (Participant 1, Manager)

"*It's not like you can refuse to go in, or ask the care to be delivered somewhere else."* (Participant 2, Manager)

"I say 'look, I'm a really bad asthmatic…do you mind if I open your front and back door just to let some of air through?' No-one has ever refused me…I have passed that [tip] on to staff I've worked with over the years." (Participant 3, Manager)

"I've had instances where people have just been getting angry and I've just had to defuse the situation by completely changing the subject, you know. I want them to know that I'm not happy, and I don't like it, but I have to do it because it's my job." (Participant 7, Care worker)

"I remember saying, you know, 'could you just not smoke while I'm in…' but people…you know, they don't see that as a problem…you know. It's their normal." (Participant 8, Care worker)

"The practicalities can be really difficult if it's someone's home…but at the same time, I have to protect staff health as well." (Participant 10, Manager)

Current strategies to reduce staff exposure to SHS during home visits

"If staff let us know that they're pregnant, then we would complete a risk assessment…and we would be highlighting that they shouldn't be around second-hand smoke…the same as if we had someone on the team who has really bad asthma or any respiratory condition." (Participant 2, Manager)

[In relation to current risk assessment guidance] *"It doesn't have anything specifically to do with exposure to second hand smoke, it's all about mental fatigue, exposure to infection, biological agents…"* (Participant 4, Manager)

[In response to the evidence base that second-hand smoke can linger in the air for up to 5 hours] *"Five hours is a lifetime to some people. They would probably throw their hands up in horror if you said, 'don't smoke for five hours'."* (Participant 8, Care worker)

"We do have a smoking policy. Um, obviously that was, um, due for review, um, August last year…but given COVID, there's a lot of the policies still to be updated." (Participant 3, Manager)

"So, when I'm assessing a client I would be saying to them about the dangers of second-hand smoke and I would be advising them, if you've got anybody in your house that smokes, ask them to go to the back door and I would be giving them hints and tips on that. It's more about protection of other family members than staff members

going into the house though." (Participant 10, Manager)

The extent to which respiratory protective equipment would offer adequate protection:

"The small blue things [masks] that you see us all wearing, they wouldn't have any benefit whatsoever. Not even for a minute." (Participant 3, Manager)

"Well we've been wearing masks at work for more than a year anyway [because of COVID-19], so… When I was in that lady's house that was a smoker, I can't say it made a great deal of difference. I could still smell it [smoke] through the mask. Because I can still smell it…I don't think it is protecting me, no." (Participant 7, Care worker)

"I don't know how much protection if offers…I've no idea." (Participant 13, Policy stakeholder)

Supporting temporary abstinence from smoking in the home prior to/during home visits

"It [NRT use] could make a big difference...They [patients] may actually realise, 'well, I don't need to have a cigarette.' And it could potentially ease a lot of the concerns that nurses have about going into smoky environments." (Participant 4, Manager)

"Few people would argue against using nicotine replacement therapy for that" [purpose] – Participant 14, Policy stakeholder).

"I would, I would welcome that [giving patients NRT for temprary abstinence from smoking in the home] I would, I would definitely welcome that… I'd love to support that, but I don't think we would ever be allowed to do that, erm, or that people [patients] would want to [use NRT in this way]. They'd be saying, 'oh no, I'd rather have a cigarette'." (Participant 7, Care worker)

"The same would happen…when we've got clients come in to hospital. You would ask them if they wanted to stop smoking. If they said 'no', that's fine, but for the period of time they're in hospital, you'll give them nicotine replacement just to curb their withdrawal symptoms." (Participant 10, Manager)

"I wouldnae really want anybody to use an e-cigarette when I'm in [their home] because I don't think there's enough knowledge about what the effects are" (Participant 9, Care worker)

"*As far as I'm concerned, I think we don't really know the dangers of they e-cigarettes yet, you know. I don't know if there's enough research to say they're, it's definitely not harmful if you're passively inhaling that. Erm, that still kind of frightens me a bit, but it's not as unpleasant as smoke."* (Participant 7, Care worker)

"I think for me, because an e-cigarette is still inhaled and exhaled, I just don't like the idea of inhaling something when I don't know if it is having any impact on my health…They don't know the long-term impact of e-cigarettes, and I do know ad hoc where people have respiratory conditions, the vape has actually exacerbated it." (Participant 13, Policy stakeholder)
